# Renal Involvement in Children with Type 2 Diabetes Mellitus Onset: A Pilot Study

**DOI:** 10.3390/children8080627

**Published:** 2021-07-23

**Authors:** Pierluigi Marzuillo, Anna Di Sessa, Pier Luigi Palma, Giuseppina Rosaria Umano, Cesare Polito, Dario Iafusco, Stefano Guarino, Emanuele Miraglia del Giudice

**Affiliations:** Department of Woman, Child and of General and Specialized Surgery, Università degli Studi della Campania “Luigi Vanvitelli”, Via Luigi De Crecchio 2, 801138 Naples, Italy; anna.disessa@unicampania.it (A.D.S.); pieropalma2710@gmail.com (P.L.P.); giusi.umano@gmail.com (G.R.U.); cesare.polito@golfonet.it (C.P.); dario.iafusco@unicampania.it (D.I.); stefano.guarino@policliniconapoli.it (S.G.); emanuele.miraglia@unicampania.it (E.M.d.G.)

**Keywords:** type 2 diabetes mellitus, acute kidney injury, renal tubular damage, children

## Abstract

Type 2 Diabetes Mellitus (T2DM) is a main cause of chronic kidney disease (CKD) in adulthood. No studies have examined the occurrence of acute kidney injury (AKI)—that enhances the risk of later CKD—and renal tubular damage (RTD)—that can evolve to AKI—in children with onset of T2DM. We aimed to evaluate the prevalence and possible features of AKI and RTD in a prospectively enrolled population of children with onset of T2DM. We consecutively enrolled 10 children aged 12.9 ± 2.3 years with newly diagnosed T2DM. AKI was defined according to the KDIGO criteria. RTD was defined by abnormal urinary beta-2-microglobulin and/or tubular reabsorption of phosphate (TRP) <85% and/or fractional excretion of Na >2%. None of the patients developed AKI, whereas 3/10 developed RTD with high beta-2-microglobulin levels (range: 0.6–1.06 mg/L). One of these three patients also presented with reduced TRP levels (TRP = 70%). Proteinuria was observed in two out of three patients with RTD, while none of patients without RTD had proteinuria. Patients with RTD presented higher beta-2-microglobulin, acute creatinine/estimated basal creatinine ratio, and serum ketones levels compared with patients without RTD. In conclusion, in our pilot observation, we found that none of the 10 children with T2DM onset developed AKI, whereas three of them developed RTD.

## 1. Introduction

Diabetes mellitus is a chronic disease that affects protein, fat, and carbohydrate metabolism [1]. There are different types of diabetes mellitus that can occur in children. The main types are represented by type 1 diabetes mellitus (T1DM) and, more rarely diagnosed, type 2 diabetes Mellitus (T2DM) [2]. T2DM is much less common than T1DM in children, accounting for a prevalence of 0.17 per 1000 among white youth [2]. Nonetheless, type 2 incidence is considerably increasing. This is probably related to the obesity and overweight epidemic increase among children [3]. Currently in the U.S., about one third of children are either overweight or obese [4]. Obesity and overweight, along with positive family history, are the main risk factors for T2DM that is characterized by hyperglycemia with normal or high insulin serum levels, related to a peripherical resistance to insulin signal [1].

The main symptoms, shared with the other types of diabetes mellitus, are polyuria, polydipsia, and polyphagia [1]. Many complications can worsen clinical course of diabetes mellitus, such as Diabetic Ketoacidosis (DKA). DKA is defined by blood glucose level ≥200 mg/dL, pH ≤7.3 or bicarbonates ≤15 mEq/L, and elevation of serum ketones [5]. Generally, the latter is more frequent in T1DM, and can even be present in up to 25% of newly diagnosed T2DM children [6].

Nephrological complications can also occur, especially in children with T1DM. Acute kidney injury (AKI) and renal tubular damage (RTD) are frequent in hospitalized patients for DKA [7,8]. Hursh et al., in their monocenter study, showed that 64.2% of the 165 retrospectively enrolled children showing DKA at the T1DM onset presented with AKI [7], according to the Kidney Disease/Improving Global Outcomes (KDIGO) creatinine criteria [9]. These data were prospectively confirmed by our research group. We showed that 65.2% of the children with DKA and 21.1% of those without DKA at T1DM onset presented AKI, while 73.5% of the children with T1DM onset presented RTD. All patients with DKA or AKI presented with RTD [8].

The RTD is characterized by a damage of renal tubular cells that determines urinary loss of solutes. AKI occurs as consequence of the RTD by vasoconstriction of the afferent arterioles, which determines fall in the glomerular filtration rate (GFR) to reduce the solutes loss in the urines. This mechanism, however, leads to a persisting renal ischemia, by resulting in acute tubular necrosis that shifts the AKI from functional to intrinsic [10].

Taking advantage of the DiAKIdney (onset of type 1 Diabetes mellitus and Acute Kidney Injury) study’s prospective data collection [8], we analyzed the data of patients admitted with the diagnosis of T2DM in the study period. We aimed to evaluate the prevalence and the possible features of AKI and RTD in children with T2DM.

## 2. Methods

Between January 2017 and July 2019, we enrolled all the children consecutively hospitalized in our Department for T2DM onset. The study was conducted accordingly with the principles outlined in the Declaration of Helsinki [11] and approved by our Research Ethical Committee. The informed consent was obtained before any procedure. The inclusion criteria were (i) onset of T2DM; (ii) age <18 years; and (iii) not having assumed any medication. Exclusion criteria were (i) denied consent; (ii) onset of other forms of diabetes; and (iii) previously known diseases of the kidney and urinary tract. None of the enrolled patients presented with present or past macroscopic/microscopic hematuria, or with history of urinary tract infection, low urinary tract symptoms, urinary calculi, or renal colic.

In all the patients, T1DM autoimmune diabetes was excluded by negativity of glutamic acid decarboxylase, islet antigen 2, insulin, and Zinc transporter 8 antibodies at the time of diagnosis.

### 2.1. Study Protocol

All the clinical evaluations and sample collections were made during the normal procedures which the newly diagnosed T2DM patients daily undergo in our center.

### 2.2. Clinical Evaluation

On enrolment, the following clinical data were collected: age, weight, height, blood pressure (BP), heart rate (HR), and urinary output for the first 6 h. We recorded if the patients at admission showed Kussmaul breathing, or were in coma, and if there was familiarity for T2DM.

Height, weight, and body mass index (BMI) with their Standard Deviation Score (SDS) were obtained as previously described [12].

BP was measured by automated oscillometric device with an appropriate cuff size. The lowest value among three measurements was taken in consideration. The BP SDS was calculated [12].

### 2.3. Biochemical Evaluation

At admission, a blood sample for the evaluation of complete blood count, creatinine, glycaemia, Na, K, Cl, blood pH, bicarbonates, ketones, and glycate hemoglobin (HbA1c), and a urine sample for urinary proteins, microalbuminuria, creatinine, sodium, calcium, phosphate, and beta2-microglobulin were collected. The urinary calcium/creatinine ratio (Uca/Ucr), urinary protein/creatinine ratio (Upr/Cr), and microalbuminuria/creatininuria ratio (Ua/Cr) were calculated. The creatinine serum levels were determined every 24 h for the first 72 h and then after 5 days from the hospitalization.

### 2.4. Definitions

#### 2.4.1. Type 2 Diabetes Mellitus

T2DM was defined by fasting plasma glucose ≥126 mg/dL (7.0 mmol/L) or 2-h plasma glucose ≥200 mg/dL (11.1 mmol/L) after oral glucose tolerance test or HbA1C ≥6.5% (48 mmol/mol) or the presence of classic symptoms of hyperglycemia plus a random plasma glucose ≥200 mg/dL (11.1 mmol/L) [13].

#### 2.4.2. Renal Tubular Damage

We considered the patient as affected by RTD if they presented with one or more abnormal values among urinary levels of beta-2-microglobulin >0.33 mg/L, tubular reabsorption of phosphate (TRP) <85%, or FENa >2% [8].

#### 2.4.3. Acute Kidney Injury

AKI was defined accordingly with the Kidney Disease/Improving Global Outcomes (KDIGO) serum creatinine and/or urine output criteria [9].

The value of creatinine estimated using previously validated back-calculation methods was considered as basal serum creatinine value [14]. In our laboratories, creatinine levels are assessed by Jaffé method; therefore, the Schwartz equation [15] was used to back-calculate basal serum creatinine, assuming that basal estimated glomerular filtration rate (eGFR) was the median age-based eGFR normative values for the children ≤2 years of age [16], and eGFR = 120 mL/min/1.73 m^2^ for children >2 years [17,18]. No AKI was defined by acute creatinine/estimated basal creatinine ratio <1.5, or by urine output >0.5 mL/kg/h, stage 1 AKI by a value of ratio from 1.5 to <2, or urine output <0.5 mL/kg/h for 6–12 h, stage 2 by a value of ration from 2 to <3, or urine output <0.5 mL/kg/h for ≥12 h, and stage 3 by a value ≥3, or urine output <0.3 mL/kg/h for ≥24 h, or anuria for ≥12 h [9].

### 2.5. Other Definitions

DKA was defined by blood glucose level ≥200 mg/dL, pH ≤7.3 or bicarbonate levels ≤15 mEq/L, and elevation of serum ketones [7,19].

The sodium levels were corrected based on glycaemia levels using the following formula [7]: corrected Na = ((Glucose (mg/dL)/18) − 5.6) × 0.36 + serum Na.

The TRP was calculated using the formula: TRP (%) = 1 − ((U_p_/P_p_) × (P_Cr_/U_Cr_)) × 100 [20].

The fractional excretion of Na (FENa) was calculated with the formula: FENa (%) = ((U_Na_/U_Cr_)/(P_Na_/P_Cr_)) × 100 [8].

Proteinuria was defined by UPr/UCr >0.2 mg/mg and microalbuminuria was defined by Ua/Cr between 30 and 300 mcg/mg [21]. Hypercalciuria was defined by UCa/Cr >0.21 mg/mg.

### 2.6. Post Hoc Power Calculation

We did not calculate a priori the sample size as we planned to enroll all the available patients. No similar studies are available in children. However, on the basis of a 43.8% prevalence of AKI in our previous study, in which the same definition was used, considering the prevalence of AKI of 0% in this population of 10 subjects, the power for AKI, with an alpha of 0.05, was 100%. Moreover, on the basis of a prevalence of 73.5% of RTD in our previous study, in which the same definitions were used, considering the prevalence of RTD of 30% in this population of 10 subjects, the power for RTD, with an alpha of 0.05, was 83.9%.

### 2.7. Statistical Analysis

*p* values < 0.05 were considered statistically significant. Differences for continuous variables were analyzed with the independent-sample t test for normally distributed variables and with the Mann–Whitney test in case of non-normality. Qualitative variables were compared by using Fisher exact test.

The Stat-Graph XVII software for Windows was used for all statistical analyses.

## 3. Results

Ten patients with T2DM were evaluated during the DiAKIdney study period. T2DM diagnosis was made in six patients due to random plasma glucose ≥200 mg/dL, and due to HbA1C ≥6.5%. All of them had the classic symptoms of diabetes, such as polyuria and polydipsia. Three additional patients had no specific symptoms (two presented with headache, one with abdominal pain and vomiting) and underwent biochemical exams due to these non-specific symptoms. These three patients presented glycemia between 140 and 194 mg/dL and the T2DM diagnosis was due to HbA1C ≥6.5%. The remaining patient was asymptomatic, and the diagnosis was accidentally made by a rapid test for glycemia measurement, showing glucose levels of 185 mg/dL with subsequent T2DM confirmation by HbA1C ≥6.5%. The rapid test for glycaemia measurement was made without any clinical indication, and only due to the availability of the glucometer at home for a parent with T2DM.

The mean age of enrolled patients was 12.9 ± 2.3 years (range: 9–14 years). Six out of 10 were of male gender. All the patients had a positive family history of T2DM. None presented AKI, whereas 3 out of 10 patients presented with RTD. Two of 10 patients (both with RTD) presented proteinuria (UPr/Cr 0.29 and 1.48 mg/mg), one (without RTD) out of 10 patients presented microalbuminuria with Ua/Cr 38 mcg/mg. Four (two with and two without RTD) out of 10 patients presented hypercalciuria (UCa/Cr range: 0.26–0.51 mg/mg).

Patients with and without RTD presented similar age (Table 1). All the patients with RTD showed high beta-2-microglobulin levels (range: 0.6–1.06 mg/L). One of these patients also presented with reduced TRP levels (TRP = 70%). Proteinuria was detected in two out of three patients with RTD, while none of the patients without RTD had proteinuria. Patients with RTD presented higher beta-2-microglobulin, acute creatinine/estimated basal creatinine ratio, and serum ketones levels compared with those without RTD (Table 1). Patients with RTD showed a clear trend for lower eGFR and TRP levels, and higher microalbuminuria, UCa/UCr, UPr/UCr, HR, and HbA1c compared with those without RTD (Table 1).

In the three patients with RTD, beta-2-microglobulin, and TRP values were re-evaluated after a variable time of 2–4 years from the T2DM onset, and these values were normal in all of them.

## 4. Discussion

Evidence in children with T1DM indicates that this condition can affect renal function both in the acute setting, at the T1DM onset, through AKI and RTD, and in the chronic setting by the diabetic nephropathy development. In the acute setting, AKI and/or RTD can manifest [7,8] with important prognostic implications. To have presented even a mild form of AKI, in fact, doubles the risk of later chronic kidney disease [22]. To have started T1DM with AKI or RTD, therefore, could significantly increase the risk of nephropathy in T1DM patients which already present, due to T1DM itself, an increased risk of diabetic nephropathy [23].

In adulthood, T2DM is one of the main causes of end stage renal disease (ESRD), accounting for 30–40% of cases in most countries. ESRD secondary to diabetic nephropathy typically manifests after 20 to 30 years of diabetes exposure [24]. In childhood, diabetes is responsible for only 0.1% of ESRD [25]. Evidence indicates, however, that renal involvement in youth onset T2DM occurs early in the disease course, and that progression is similar to that seen in adult T2DM onset [26]. Early manifestation of T2DM renal complication is represented by microalbuminuria, the most commonly reported complication in children and adolescents with T2DM [27]. Estimates vary widely between 7 and 22% at presentation, and between 9.6 and 72% within 3–10 years after diagnosis [25]. In line with these findings, we found proteinuria and/or microalbuminuria in 3 out of 10 patients.

Our pilot study aimed to investigate the effect of T2DM onset on renal function in childhood. To date, no other studies have specifically evaluated the prevalence of AKI and RTD in a population of children with onset of T2DM. Interestingly, we found that none of the patients with T2DM onset developed AKI. This data is significantly different from the evidence in children with T1DM onset, which presented AKI in up to 65% of cases [7,8]. In the current study, despite 6 out of 10 patients with T2DM onset presenting polyuria and polydipsia, none developed AKI, probably as, in the setting of T2DM, the polyuria is lesser in degree compared with T1DM setting, where the lack of insulin and the osmotic diuresis could be massive with subsequent increased risk of hypovolemia.

On the other hand, 3 out of 10 patients with T2DM onset presented a RTD with beta-2 microglobulin increase in all the cases and with TRP reduction in one of them. Again, this data is less alarming comparing with T1DM patients which can present RTD in up to 73.5% of the cases [8]. In our opinion, the fact that 3 out of 10 patients with T2DM onset presented a RTD is relevant as, in the AKI pathophysiology, the RTD represent the first step of damage progression. In fact, when hypovolemia occurs, an RTD establishes itself with subsequent failure to reabsorb filtered solutes [10]. To compensate for this solutes loss, a renal vasoconstriction determines an adaptive fall in the glomerular filtration rate, thus determining AKI [10]. However, if ischemia persists, the damage becomes heavier, and the AKI could shift from functional to intrinsic (acute tubular necrosis) [28,29]. The fact that the RTD did not evolve to AKI in our pilot cohort could be linked to the fact that, in all the patients, the T2DM has been diagnosed before deterioration of general conditions (hyperglycemic crisis). This could be related to the T2DM family history in all the patients. Indeed, this latter could have determined higher awareness of the symptoms of T2DM in families, facilitating an early diagnosis. This could be indirectly supported by the evidence of the DiAKIdney study showing that patients with family history of T1DM showed a significantly shorter duration of polyuria and polydipsia, and lower prevalence of the AKI, acute tubular necrosis, and RTD, when compared with patients without any family history [8]. Another explanation could be that the T2DM onset is not so severe as in case of T1DM, with subsequently lesser renal involvement. In fact, in the T1DM pathophysiology, the hyperglycemia abruptly develops when 80–90% of pancreatic beta cells are destroyed by the autoimmune process. This determines the onset of severe polyuria and polydipsia, with important dehydration and secondary heavy kidney involvement evolving to AKI, and acute tubular necrosis if the insult persists [7,8,30]. On the other hand, in the T2DM pathophysiology, the development of alterations in glucose metabolism results from the gradual fall in beta cell function occurring within a background of insulin resistance [31]. The transition from prediabetes to T2DM is usually a gradual phenomenon [31], and, in this case, the acute damages of hyperglycemia are limited, and therefore the evolution of RTD to AKI is less likely. Moreover, the transition from prediabetes to T2DM being a gradual phenomenon, the hypovolemia at T2DM onset is also milder than that at T1DM onset, and therefore the RTD in T2DM could be milder.

The number of children with T2DM limited our possibilities of analysis. Despite the limited patients’ number, we found that patients with RTD at T2DM onset presented higher serum ketones and acute creatinine/basal creatinine ratio levels than patients without RTD at T2DM onset (Table 1). This data could give, however, interesting pathophysiological information. The RTD, in fact, could also be linked to the acidosis degree if the patients do not develop overt diabetic ketoacidosis, and to the degree of creatinine levels increase compared with creatinine basal levels, also if the creatinine increase (expressed as acute creatinine/basal creatinine ratio) does not reach the KDIGO definition of AKI [9]. This is in line with the data available in children with T1DM onset in which similar associations with AKI [7,8] and RTD were found [8], in patients both with and without DKA at T1DM onset.

The limited number of enrolled patients did not allow us to make a logistic regression analysis for identification of RTD risk factors. Therefore, this is only to be considered a pilot study, opening the way to future multicenter studies. The second limitation is the lack of standardized follow-up visits to precisely determine the timing of normalization of the renal tubular function. In fact, in the three patients with RTD, the beta-2-microglobulin and TRP levels were re-evaluated after a range of 2–4 years from T2DM onset. However, in children with T1DM onset, the renal tubular function normalized in all patients within 2 months [8] and the possibility exists that the renal tubular function also normalizes in children with T2DM within this temporal range. The third limitation is that we defined RTD only on urine sample at admission. However, we determined the creatinine serum levels every 24 h for the first 72 h, and then after 5 days from the hospitalization, therefore we can affirm with certainty that none of the patients developed a tardive AKI during hospitalization, and that none of the patients with RTD progressed towards AKI. Moreover, as the onset of T2DM is milder than T1DM, it is unlikely that a patient, not having developed RTD before hospitalization, develops RTD after admission, and after proper management of T2DM has started.

The last limitation is represented by the lack of information about levels of cystatin C, neutrophil gelatinase-associated lipocalin, and alfa-1-microglobulin in plasma and urine, as these are common markers for kidney injury.

A strength of this study is the prospective data collection that allowed an extensive study of the other markers of glomerular and tubular renal function.

In conclusion, in our pilot observation, we found that none of the 10 children with T2DM onset developed AKI, whereas three of them developed RTD. Future large studies on this topic are needed.

## Figures and Tables

**Table 1:** Clinical and biochemical characteristics of all the enrolled patients and of the patients with and without RTD at T2DM diagnosis.

	All Patients No. = 10	RTD (Yes) No. = 3	RTD (No) No. = 7	*p*
Age, yr	12.9 ± 2.3	11.2 ± 3.1	13.8 ± 1.6	0.11
Male sex, No.	6	2	4	0.99
T2DM familiarity, No.	10	3	7	0.99
BMI, SDS	5.2 ± 3.2	4.1 ± 1.5	5.6 ± 3.7	0.53
SBP, SDS	1.3 ± 1.2	1.6 ± 1.7	1.1 ± 1.1	0.58
DBP, SDS	0.8 ± 0.9	0.9 ± 1.2	0.7 ± 0.8	0.73
HR, beats/min	91.7 ± 24.9	104.3 ± 27.5	86.1 ± 16.9	0.22
HR > 2SDS for age and gender, No.	2	1	1	0.56
Kussmaul, No.	0	0	0	0.99
Coma, No.	0	0	0	0.99
DKA, No.	0	0	0	0.99
Serum bicarbonate level, mEq/L	26.6 ± 10.7	21.2 ± 4.4	28.9 ± 11.9	0.31
Serum ketones, mmol/L	1.4 ± 1.9	3.9 ± 1.1	0.34 ± 0.69	<0.0001
AKI, No.	0	0	0	0.99
eGFR, mL/min/1.73 m2	133.3 ± 34.5	112.76 ± 17	142.1 ± 32.3	0.23
Serum creatinine, mg/dL	0.69 ± 0.08	0.69 ± 0.08	0.69 ± 0.09	0.88
UPr/Cr *, mg/mg	0.29 ± 0.42	0.63 ± 0.74	0.14 ± 0.04	0.09
Microalbuminuria *, mg/L	15.8 ± 11.8	20 ± 11.8	6 ± 1	0.08
Ua/Cr *, mcg/mg	15.9 ± 8.4	18.0 ± 9.3	11.1 ± 3.3	0.26
UCa/Cr *, mg/mg	0.18 ± 0.16	0.32 ± 0.22	0.12 ± 0.1	0.08
TRP *, %	91 ± 8.3	85.6 ± 14	93 ± 4	0.2
Beta2-microglobulin *, mg/L	0.27 ± 0.27	0.76 ± 0.26	0.06 ± 0.05	<0.0001
Corrected serum Na level, mEq/L	141.3 ± 2.8	142.5 ± 4.8	140.7 ± 1.6	0.41
Serum phosphorus level, mg/dL	3.9 ± 0.05	3.7 ± 0.73	3.9 ± 0.4	0.54
Serum chloride levels, mEq/L	102.0 ± 3.7	103.3 ± 1.5	101.5 ± 3	0.34
HbA1c, %	10.2 ± 2.31	11.9 ± 2.4	9.5 ± 2.1	0.16
Acute Creatinine/Basal Creatinine	0.99 ± 0.17	1.2 ± 0.2	0.9 ± 0.6	0.006
FENa *, (%)	0.66 ± 0.22	0.70 ± 0.1	0.6 ± 0.3	0.9

For continuous variables, means ± SDS are shown. Abbreviations: AKI, acute kidney injury; BMI, body mass index; DBP, diastolic blood pressure; DKA, diabetic ketoacidosis; eGFR, estimated glomerular filtration rate; FENa, fractional excretion of Na; HbA1c, glycate hemoglobin; HR, hearth rate; SBP, systolic blood pressure; SDS, standard deviation score; T2DM, type 2 diabetes mellitus; TRP, tubular reabsorption of phosphate; Ua/Cr, microalbuminuria/creatininuria ratio; UCa/Cr, urinary calcium/creatinine ratio; and UPr/Cr, urinary protein:creatinine ratio; * Measured on urine samples collected at hospitalization.

## Data Availability

Data supporting reported results can be obtained on request.
